# Ranging Patterns of Critically Endangered Colobine, *Presbytis chrysomelas chrysomelas*

**DOI:** 10.1100/2012/594382

**Published:** 2012-03-12

**Authors:** Ahmad Ampeng, Badrul Munir Md-Zain

**Affiliations:** School of Environmental and Natural Resource Sciences, Faculty of Science and Technology, Universiti Kebangsaan Malaysia, Selangor, 43600 Bangi, Malaysia

## Abstract

*Presbytis chrysomelas chrysomelas* endemic only in Sarawak and Kalimantan was categorized by IUCN as a critically endangered primate that require special attention from research and conservation perspectives. A qualitative study on ranging patterns of *P. c. chrysomelas* was conducted in the Samunsam Wildlife Sanctuary, Sarawak. The study was conducted over a period of 13 months from December 2004 to December 2005 with 213 days of observation. Behavioural observation covered 17 groups with special emphasis on two main groups and 1 subadult group. Scanning and focal sampling were employed as the observation methods. Results indicated that *P. c. chrysomelas* had vertical, straight horizontal, and cross-horizontal types of movement patterns. *P. c. chrysomelas* was recorded to have a short movement distance (31.8–54.3 m). Distribution, abundance types, and food resources might be the factors that shaped the patterns of movement and distance in *P. c. chrysomelas*.

## 1. Introduction

*Presbytis chrysomelas chrysomelas* is classified by the IUCN as a critically endangered species identified as a member of the family Cercopithecinae, subfamily Colobinae [1]. *P. c. chrysomelas* can only be found in Sarawak and Kalimantan as among the Malaysian *Presbytis* species along with* P. hosei, P. rubicunda, P. frontata, *and* P. melalophos* [2]. Previously, it has been classified as a subspecies of the *Presbtis melalophos* [3, 4]. However, Groves [1] categorized the species based on morphological data, and his finding was supported by molecular systematic data [5]. Bennett [6] noted that the only three locations available for *P. c. chrysomelas *in Borneo are the Samunsam Wildlife Sanctuary (SWS), the Similajau National Park in Sarawak, and the Ulu Sg. Kapuas in West Kalimantan, Indonesia. In addition, Ampeng [7] further reported the distribution of this langurs at Tanjung Datu National Park, Sarawak. *P. c. chrysomelas *is among four species of the genus *Presbytis* distributed in Borneo [8]. *P. c. chrysomelas* ecological and behavioral aspects are less known than those of *P. frontata *[9]. Many studies involving the Borneo colobines focused on *P. hosei* [10–12] and *P. rubicunda *[13, 14]. Meanwhile, other primate surveys have been conducted outside Borneo involving other *Presbytis* species such as *P. comata* [15], *P. melalophos* [16, 17], and *P. thomasi* [18, 19]. 

Ranging patterns in many primates are influenced by ecological, behavioral, and climate factors such as rainfall [20–22]. The availability and spatial patterning of food resources [23], group size [24–26], mating opportunity [27], sleeping site [28, 29], and parasite avoidance [30] are among the factors affecting the ranging patterns in primates. Many studies have attempted to explain ranging patterns of Southeast Asian colobines [11, 16, 31]. However, the study on ranging patterns in *P. c. chrysomelas* has yet to be conducted. No intensive study is reported on the behavioural aspect of this species thus causing the lack of understanding in the behavioural ecology of the colobines species in Sarawak. In this paper, qualitative assessments were done on the ranging behavior of the *P. c. chrysomelas* in the Samunsam Wildlife Sanctuary, Sarawak, by describing types of movement and daily distance travel and discovering the potential factors influencing their ranging behavior. 

## 2. Methods

**Figure 1:**
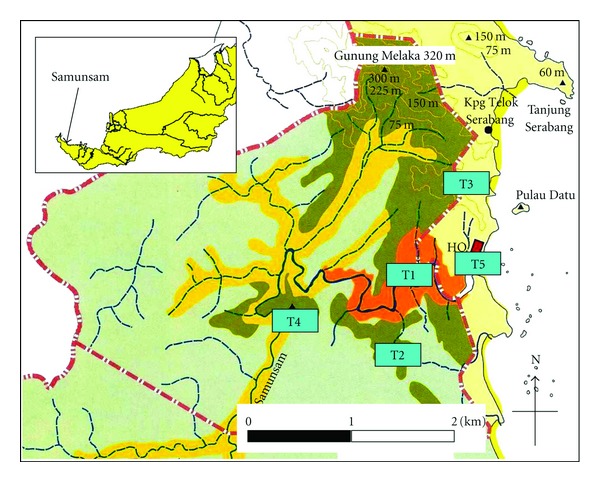
Map of the Samunsam Wildlife Sanctuary to indicate area of line transects (T1–T5).

This study was conducted at the Samunsam Wildlife Sanctuary, Sarawak, Malaysia, (SWS: 1° 78′ N and 109° 36′ E) with the area size of 60.9 km² (6092 ha). The Samunsam Wildlife Sanctuary (Figure 1) was originally established for the conservation of *Nasalis larvatus *[6, 32]. The sanctuary comprised of five habitats, namely, the riverine forest, mangrove forest, tropical heath forest, secondary forest, and lowland mix dipterocarp forest [6, 33]. However, in this study, the division was expanded into six habitats by differentiating hill mix dipterocarp from the hilly part of lowland mix dipterocarp forest. The division was based on the discovery of *Shorea curtisii* in the hilly part of the sanctuary. According to Hamid [34], *S. curtisii* can be used as the main indicator for the occurrence of hill mix dipterocarp forest. The Samunsam Wildlife Sanctuary was under the jurisdiction of the State Government of Sarawak (Sarawak Forest Department). However, since 2003, this sanctuary was supervised by the management of the Sarawak Forestry Corporation Sendirian Berhad (SFCSB). 

One transect line of 2 km length has been set in the hill dipterocarp forest, lowland dipterocarp forest, riverine forest, tropical heat forest, and 1 km long in the secondary forest (Figure 1). Transects were surveyed and categorized commonly from time of dawn (06:00–06:30) until dusk (17:00–18:30). Detecting groups were discussed in detail by Ampeng and Md-Zain [35]. Within a 13-month study period (November 2004 to December 2005), 17 groups of *P. c. chrysomelas* were encountered in the SWS (Table 1). Out of this, nine groups were identified to originate from the main groups (MG) and eight were from the subadult groups (SAG). Main and subadult groups were defined based on age status. All groups were observed but only three groups were constantly followed during the study. The forested habitat and the arboreal habits of the *P. c. chrysomelas* made the groups extremely difficult to follow. The illegal logging and hunting, weather condition, and the shyness of the monkeys also limited the result of the survey. Three groups were focused on, two main groups (MG) and a subadult group (SAG). MG group and SAG in transect three and MG in transect two were relatively tolerant to the presence of the researchers. 

Scanning sampling was employed during intensive observation [36–38]. Every sampling subsession was 15 minutes with 10 minutes allocated for scan sampling and 5 minutes for describing and concluding each subsession. Langurs in the Samunsam Wildlife Sanctuary were not habituated in the presence of humans because of poachers who often enter the area [39]. To overcome the problem, focal sampling was also used as an alternative to record langur behavior [40, 41]. Focal sampling was employed each time an individual was seen; only one individual was observed at a time. In this study, quantitative ranging pattern ethogram was scored based on Fashing [22]. Furthermore, in this paper, further details to explain the types of langur movements throughout the study period were provided based on qualitative assessment. Daily distance movement based on Rabinowitz [40] were measured, and potential factors that shape types and distant movement of *P. c. chrysomelas* in Samunsam Wildlife Sanctuary were explored. The collected data were not analyzed using statistical methods as data acquisition required more detailed qualitative description [42]. 

## 3. Results

### 3.1. Movement Patterns

*P. c. chrysomelas* was not found to be dispersed or scattered during daily activities, especially when feeding the young leaves or fruits. This movement pattern was used as the main indicator to distinguish the group *P. c. chrysomelas* with the other diurnal primates found in this sanctuary. Observation showed three types of movement patterns; vertical, straight horizontal, and cross-horizontal movements. 

**Figure 2:**
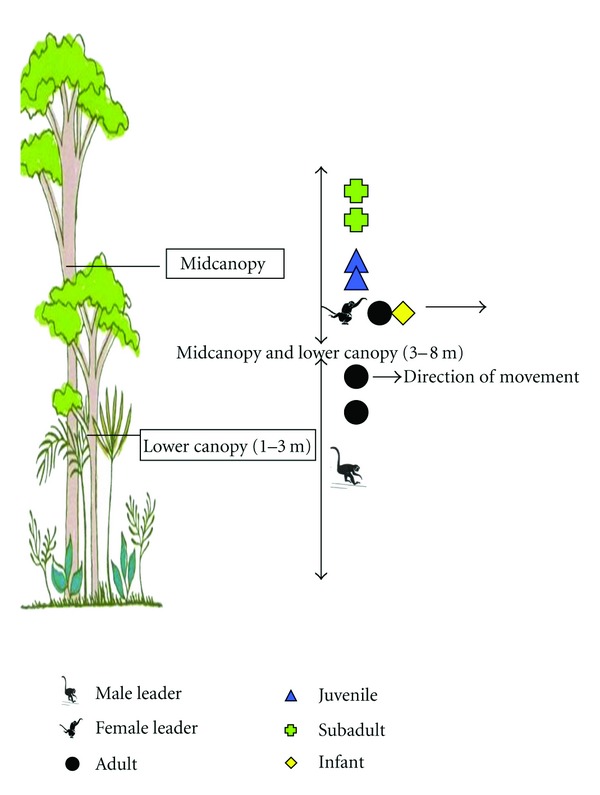
Illustration of vertical movement practiced by the main groups at mid- and lower canopy.

Vertical movement was practiced only by the main groups (Figure 2). It was observed in the morning and not during the afternoon. The main groups moved in the vertical mid-canopy and lower canopy (3–8 m). This movement was consistent with the male leader in the bottom position (1–3 m) and performed only from March to July 2005. 

**Figure 3:**
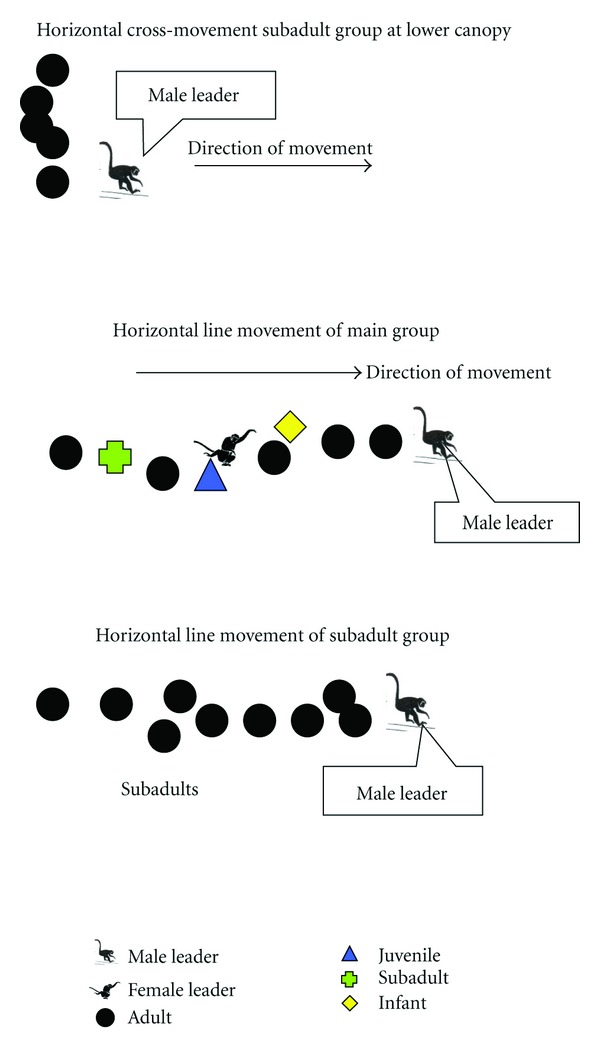
The horizontal cross- and horizontal line movement of *P*.* c*. *chrysomelas. *

For other months beyond March to July 2005, the pattern of movement of the main groups and subadults was in a horizontal line at midcanopy level. At this time, the young leaves and fruit resources were abundant in the mid-canopy level. This is similar to findings by Bourliere [43] and Napier [44] who found that the pattern of movement was associated with the use of canopy space. The straight horizontal movement pattern was common to both groups during the beginning of their daily activities. For main groups, the adult male was observed to be positioned in front to lead the movement. Adult females were observed to move in the middle of the group with the infant, juvenile 1 and juvenile 2. Subadults moved in the back position. Some adults (1-2 individuals) travelled at the second and third positions (Figure 3). For the sub-adult group, the male leader moved forward once while others followed behind (Figure 3). 

Distribution of groups to the smaller units during foraging was not recorded in the main nor sub-adult groups. However, when fruit resources were available (the size of trees ≤30 cm), rotation on trees occurred between individuals of the main groups. Only 2-3 individuals were on the trees at one time consuming fruits. Meanwhile, others waited on adjacent trees (50–10 m), consuming leaves or watching the surrounding. Afterwards, the first group would leave to wait in nearby trees (50–10 m) and be replaced by a group of 2-3 individuals who were yet to eat. It was only after everyone finished eating fruits that all individuals in the group would travel together. This pattern of behavior, however, was only recorded twice in the main group. The results showed that both main and sub-adult groups moved towards the limit of the temporary home range if the group moved in a straight horizontal pattern. The results of this study agreed with studies by Lakim [11] and Fashing [22] in which the movement pattern was determined by the distance of daily movement, finding of food sources, and determination of the home range limit. 

During horizontal movement, a low and short vocal tone was produced. A short vowel sound was produced during horizontal movement in determining the size of the home range area. The group would respond to this sound by turning either left or right. The results of this study agreed with those by Boonratana [45] who observed a straight horizontal movement in order to determine the limits of the home range. Straight horizontal movement was used by both groups while moving in the middle of the canopy space. The tree branches in the middle of the canopy space were large enough to accommodate the movement of individuals and also up to 2–5 individuals at a time. 

The horizontal cross-movement was observed for the sub-adult group when travelling in the lower canopy (1–3 m) (Figure 3). This movement pattern was recorded only to occur between the months of March to July 2005 and involved 25% of the total movement recorded. This movement pattern only occurred in the morning. For the sub-adult group, the pattern of horizontal movement contradicted the vertical movement pattern of the main group. The space under the canopy tree size was small with limited food resources. This situation made it difficult for individuals to be at one tree at a given time as others may have already occupied it. 

### 3.2. Daily Movement Distance

Daily movement distance for the whole period observation between the main groups and sub-adult group was different. For main groups, the total hours of daily movements spent from morning to late evening was 448.3 hours with a total movement distance of 11,344.7 m. In the morning, the total number of hours of movement observed for the main groups was 243.73 hours with an average of 1.44 ± 0.03 hours. With a total movement distance of 5978.5 m in the morning, the estimated daily movement distance was 35.4 ± 0.7 with a range between 33.9 and 36.8 m and speed of movement of about 27.2 m/hr. In the evening, the total number of hours of movement for the main groups was 204.54 hours with an average of 1.2 ± 0.04. With a total movement distance of 5366.2 m, the estimated range of daily movements in the afternoon was 31.8 ± 0.6 with a range of 30.5–33 m and a speed of about 26.5 m/hr (Table 2). 

For the sub-adult group movement, a total number of 287.23 hours was spent in the morning and evening with a total movement distance of 4329 m. In the morning, the movement for the sub-adult group was observed to be 150.64 hours with an average of 3.42 ± 1.30 hours and a movement distance of 2386.8 m. The average distance of movement in the morning was 54.3 ± 9.1 m with a speed of about 16 m/hr. In the evening, 136.59 hours of the movement was observed for the subadult group, with an average 3.1 ± 0.9 hours. Total distance movement observed in the evening was 1942.2 m. The average distance of movement in the afternoon was 44.1 ± 1.4 m with a speed of about 14 m/hr (Table 2). 

## 4. Discussion

Distribution and abundance of food resources in the Samunsam Wildlife Sanctuary may influence the pattern and range movement of *P. c. chrysomelas*. Main and sub-adult groups of *P. c. chrysomelas* entered temporary home range when fruit sources were available. This is in agreement with studies by Fashing and Cords [46] and Fashing [47] which stated that the distribution and abundance of food resources is a major factor influencing the ranging pattern of primates. These findings also concurred with Zhang and Wang [48] who observed that food resources, especially from abundant leaves affected the daily movement range of primates. 

When food is scarce, the distance of daily movement may be increased [16, 49]. This contradicted findings in this study; *P. c. chrysomelas* did not seem to suffer food shortage despite a short daily movement distance. The results of this study supports Fashing [22] who observed a close distance movements of many leaf monkeys. 

Ostro et al. [25] found that group size can influence the daily movement distance. Daily movement distance of the big group size is greater than that of the small group size [50]. This was observed in this study since the group size of main group is usually greater than that of sub-adult group. Group size of *P. c. chrysomelas* in the Samunsam Wildlife Sanctuary is small, between 8 and 13 individuals. With a small group size and food sources abundant in the Samunsam Wildlife Sanctuary, groups do not need to forage in long distance. 

The use of different habitats will influence the pattern and range of leaf monkey movement [33]. The use of different habitats in the Rajanathan [33] study was based on the availability of fruit resources. This statement is true for this study because *P. c. chrysomelas* entered different habitats in the temporary home range when there were fruit resources. *P. c. chrysomelas* moved far only when fruit sources were available. Fruit trees in the forest habitat of the Samunsam Wildlife Sanctuary were scattered. This caused *P. m. chrysomelas* to move a little further to get those fruits. This study supports the findings of Zhang and Wang [48] and Fashing [22] which found that the long daily movement distance is influenced by the presence of fruit trees. Observations of* P. siamensis melalophos *in the Universiti Kebangsaan Malaysia also showed that the daily movement distance was influenced by the availability of fruit resources. 

## Figures and Tables

**Table 1:** Group composition of *P. c. chrysomelas* in the Samunsam Wildlife Sanctuary, (December 2004–December 2005) (MG = main group, SAG = subadult group). Three groups (italic) were consistently followed during research period.

Transect	Group type	Adult/subadult		Juv. 1	Juv. 2	Subadult	Individual count, Dec. 04-Jan. 05	Born Infant Jan-Mar. 2005	Individual count, Dec. 31 2005
		MG	SAG						
1	MG	5	—	1	1	2	9	1	9
	MG	6	—	—	1	2	9	1	9
	SAG	—	2	—	—	—	2	—	7
	SAG	—	5	—	—	—	5	—	9

2	* MG*	* 7*	—	* 1*	* 2*	* 3 *	* 13*	* 1*	* 13*
	SAG	—	7	—	—	—	7	—	8
	SAG	—	6	—	—	—	6	—	9

3	MG	5	—	1	1	2	9	1	9
	MG	6	—	—	2	2	10	1	10
	MG	4	—	1	1	2	8	1	8
	*MG*	*6*	*—*	*1*	*2*	*2*	*11*	*1*	*11*
	SAG	—	5	—	—	—	5	—	8
	SAG	—	7	—	—	—	7	—	9
	*SAG*	*—*	*8*	*—*	*—*	*—*	*8*	*—*	*9*

4	MG	6	—	—	—	3	9	1	9
	MG	—	5	1	1	3	9	1	9
	SAG	—	8	—	—	—	8	—	12

**Table 2:** Daily movement of main and subadult group of *P. c. chrysomelas. *

Period	Distance (m)	Standard deviation	Range	Speed (m/hour)
Main group				
Morning	35.4	0.7	33.9–36.8	27.2
Evening	31.8	0.6	30.5–33	26.5

Sub-adult group				
Morning	54.3	1.9	50.3–58.2	16
Evening	44.1	1.4	41.3–47	14
